# Remote sensing-based time series models for malaria early warning in the highlands of Ethiopia

**DOI:** 10.1186/1475-2875-11-165

**Published:** 2012-05-14

**Authors:** Alemayehu Midekisa, Gabriel Senay, Geoffrey M Henebry, Paulos Semuniguse, Michael C Wimberly

**Affiliations:** 1 Geographic Information Science Center of Excellence, South Dakota State University, Brookings, SD, 57007, USA; 2 US Geological Survey Earth Resources Observation and Science (EROS) Center, Sioux Falls, SD, USA; 3 Health, Development, and Anti-Malaria Association (HDAMA), Addis Ababa, Ethiopia

**Keywords:** Malaria, Early warning, Early detection, Remote sensing, Climate, Time series model, Forecast

## Abstract

**Background:**

Malaria is one of the leading public health problems in most of sub-Saharan Africa, particularly in Ethiopia. Almost all demographic groups are at risk of malaria because of seasonal and unstable transmission of the disease. Therefore, there is a need to develop malaria early-warning systems to enhance public health decision making for control and prevention of malaria epidemics. Data from orbiting earth-observing sensors can monitor environmental risk factors that trigger malaria epidemics. Remotely sensed environmental indicators were used to examine the influences of climatic and environmental variability on temporal patterns of malaria cases in the Amhara region of Ethiopia.

**Methods:**

In this study seasonal autoregressive integrated moving average (SARIMA) models were used to quantify the relationship between malaria cases and remotely sensed environmental variables, including rainfall, land-surface temperature (LST), vegetation indices (NDVI and EVI), and actual evapotranspiration (ETa) with lags ranging from one to three months. Predictions from the best model with environmental variables were compared to the actual observations from the last 12 months of the time series.

**Results:**

Malaria cases exhibited positive associations with LST at a lag of one month and positive associations with indicators of moisture (rainfall, EVI and ETa) at lags from one to three months. SARIMA models that included these environmental covariates had better fits and more accurate predictions, as evidenced by lower AIC and RMSE values, than models without environmental covariates.

**Conclusions:**

Malaria risk indicators such as satellite-based rainfall estimates, LST, EVI, and ETa exhibited significant lagged associations with malaria cases in the Amhara region and improved model fit and prediction accuracy. These variables can be monitored frequently and extensively across large geographic areas using data from earth-observing sensors to support public health decisions.

## Background

Malaria is one of the leading public health problems in most of sub-Saharan Africa, particularly in Ethiopia. Almost 75% of the landmass of Ethiopia is estimated to be affected by malaria, and about 68% of the population of Ethiopia (approximately 52 million people in 2007) is at risk of malaria corresponding to the human population living in areas below 2000 m elevation [1-3]. Malaria transmission is seasonal in Ethiopia and varies across the country depending on climatic and ecological factors favourable to disease-transmitting vector and parasite development, including elevation, rainfall, and temperature [1,3,4]. The major malaria transmission season occurs primarily during September to December following the main rainy season, which occurs from June to September with peak precipitation in July and August [1-4]. Large-scale malaria epidemic outbreaks occur particularly in the Ethiopian highlands where transmission is unstable, immunity of the population is low, and almost all age groups of the population are at risk of severe morbidity and mortality from malaria [1,3]. In Ethiopia epidemics occur in 5–8 year cycles; several epidemics have been reported in recent years, for example, in 2003 and 2005 [1,2]. Accordingly, there is a pressing need to develop malaria early warning to enhance public health decision making for control and prevention of malaria epidemics.

A malaria early-warning system that provides predictions of the temporal and spatial pattern of epidemics could help to control and prevent malaria epidemics. If an effective malaria early-warning system were operational, it could assist public health decision makers in prioritizing scarce resources to areas and periods most at risk [5-7]. One approach to developing a malaria early-warning system is to use statistical forecasting models based on historical malaria cases and environmental risk indicators. Thus, quantifying the links between meteorological and environmental variables and malaria is a key step towards effective malaria early warning. However, a major limiting factor is the lack of adequate environmental data covering the spatial and temporal variability of key environmental risk factors. The availability of spatially extensive and temporally consistent data from earth-observing sensors offers a source of environmental information for the development of epidemiological forecasting models. Measurements of the spatial and temporal patterns of environmental conditions that influence parasite development and the mosquito life cycle can be obtained by using remotely sensed data [8].

The influences of climatic variables on malaria risk have been well documented in previous research. Temperature, rainfall, and humidity are key determinants for malaria risk. Rainfall is associated with the presence and persistence of pools for the female anopheline mosquitoes to lay their eggs and for the completion of larval development [5,9]. Although rainfall is necessary to produce breeding sites for the mosquito to complete its life cycle, excessive rainfall can have an adverse effect by washing away mosquito larvae [10]. Temperature is associated with the durations of larval development, mosquito survival, and parasite development [5,9]. Humidity is associated with the persistence of the breeding sites and mosquito survival. Moreover, vegetation is not only a surrogate variable for the presence of moisture, but also provides a resting site for adult mosquitoes. Previous efforts to develop malaria early warning in sub-Saharan Africa have tried to link temporal patterns of morbidity with climatic variability [11-13]. Studies in the East African highlands have demonstrated significant lagged associations of temperature and rainfall with malaria cases [14,15]. The current research will expand on these studies to examine the temporal variability of malaria risk in response to satellite-derived meteorological and environmental variables in the Ethiopian highlands.

In Ethiopia, only a few prior studies quantified the effects of environmental variables on malaria risk [5,13,16,17]. A common limitation of these studies is their reliance on meteorological station data alone. Meteorological stations tend to be sparsely distributed in developing countries [18]; thus, it is difficult to obtain meteorological and environmental variables that span larger geographic areas. Earth observation data from space-borne sensors with high temporal resolution (daily to weekly) can provide alternative sources of information on meteorological and environmental variability [9,18]. Despite the advances in terrestrial remote sensing over the past three decades, only recently have these data been widely applied to tackle problems in public health [9,19-22].

Remote sensing-derived environmental variables, including indices like the normalized difference vegetation index (NDVI), the enhanced vegetation index (EVI), and land surface temperature (LST), have been used to quantify vector-borne disease risk and for malaria early warning [8,9,19,23,24]. Satellite-based precipitation measurements along with vegetation indices and LST have been shown to be useful for malaria prediction [9,24]. However, there are other types of remotely sensed environmental variables, such as actual evapotranspiration (ETa), that have not yet been explored in the context of malaria early warning. ETa measures the flux of moisture at and below the soil surface that transpires through plants and into the atmosphere as water vapour and the moisture at the surface that directly evaporates into the atmosphere [25]. Because ETa is limited by water availability, a high ETa is often indicative of high levels of surface water and soil moisture. Thus, remotely sensed estimates of ETa may indicate suitable environmental conditions for mosquito breeding and larval growth and development.

To date, there has not been a study examining the direct application of remote sensing data to model malaria risk in Ethiopia. Thus, the research presented here will help to fill this knowledge gap by (1) quantifying the relationships between satellite-derived climatic and environmental variability and temporal patterns of malaria risk and (2) developing a model of malaria risk that incorporates both historical surveillance and environmental data. In this study the following research questions were addressed. First, are remotely sensed variables significantly associated with temporal patterns of malaria risk? Second, what are the temporal lags at which each environmental variable is associated with malaria risk? Third, does the addition of remote sensing covariates improve time series model fits compared to models based only on historical case data? A time series modelling approach, which has been widely used in previous research to quantify associations between environmental variability and disease risk, was used to answer these questions [5,9,14,16,26].

## Methods

### Study area

**Figure 1:**
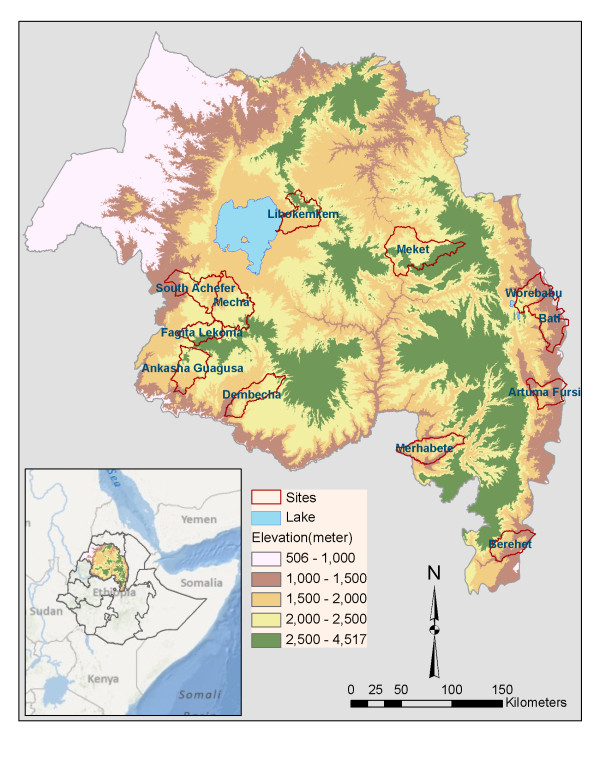
**Location of study sites: 12 districts (*woredas*) in the Amhara region of Ethiopia.**

The Amhara region is located in the north-western and north-central parts of Ethiopia and lies between 9.0° and 13.75° N and 36.0° and 40.5° E (Figure 1). The region has a population of nearly 17 million according to the 2007 census. Almost 89% live in rural households practicing subsistence agriculture. Elevation ranges from 506 to 4,517 m above sea level. The major malaria transmission season is September through December while a shorter transmission season occurs from April to May [1]. The major rainy season is typically from June to September [1,4]. Average annual temperature ranges from 16 to 27°C. The mean annual rainfall ranges from 770 to 2,000 mm.

### Data source

#### Surveillance data

The malaria cases used in this study are reports of clinically diagnosed outpatients from 12 district (*woreda*) health centres in the Amhara region of Ethiopia. These health facilities provide medical service primarily to the patients living in that district as well as some patients coming from nearby districts. Historical surveillance data were collected by visiting individual health centres to obtain hard copies of historical surveillance data and converting these data into digital format. The integrated disease surveillance and response (IDSR) data format was the source of information used in the data collection process. Although there is a recent effort by the Ministry of Health of Ethiopia to upgrade health data collection and management into a health management information system framework, there is at present no centralized database for malaria case data. The 12 districts used in this study had complete monthly counts of outpatient malaria cases for the period 2001–2009.

#### Environmental data

Satellite-derived daily rainfall estimates were obtained from the Tropical Rainfall Measuring Mission (TRMM). TRMM is a joint mission by NASA and the Japan Aerospace Exploration Agency (JAXA) to monitor precipitation in tropical and subtropical regions of the globe. The daily TRMM product (3B42), which has a spatial resolution of 0.25° x 0.25°, was extracted using the Giovanni system in the NASA Goddard Earth Sciences Data and Information Services Center (GES DISC). Monthly accumulated rainfall (mm) was summarized for each district from daily rainfall estimates.

Vegetation indices and LST were from the moderate resolution imaging spectroradiometer (MODIS) instruments on-board the Terra and Aqua satellites, which have overpass times at 10.30/22.30 and 13.30/01.30, respectively. These sensors acquire data in 36 spectral bands ranging from visible through thermal infrared. The MODIS Terra LST product (MOD11A2), which is an eight-day composite with a 1 km spatial resolution, was used for this research. Mean monthly LST (°C) was computed for every district as the average of daytime (10.30) and night-time (22.30) LST retrievals.

The MODIS Nadir BRDF-Adjusted Reflectance product (MCD43B4), which is a 16-day rolling composite updated every eight days with a 1 km spatial resolution, was used to compute the monthly mean of two vegetation indices, NDVI and EVI, for every district. NDVI and EVI were calculated from surface reflectance in the red, near-infrared, and blue bands that provides information about the greenness of the vegetation canopy. The MCD43B4 product was used to generate these vegetation indices instead of the already processed MODIS vegetation indices because this product combines multiple views from the Terra and Aqua satellites and uses Bidirectional Reflectance Distribution Function (BRDF) models to provide more consistent measurements of surface reflectance. The MODIS actual ETa product (MOD16A2), which is an eight-day composite with a 1 km spatial resolution, was used to summarize monthly mean daily ETa (mm) for every district.

### Data analysis

**Figure 2:**
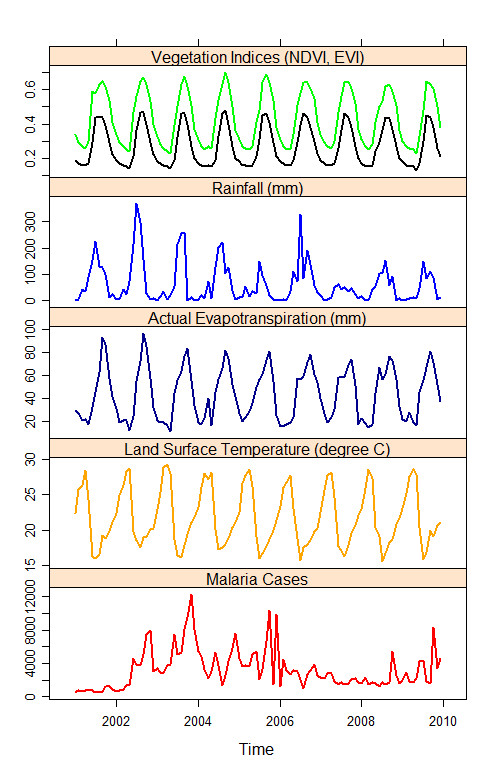
Time series for Mecha district of (from top to bottom) average monthly NDVI (normalized vegetation index) in green and EVI (enhanced vegetation index) in black (dimensionless) summarized from sixteen-day composites, total monthly rainfall (mm) summarized from daily rainfall, total monthly actual evapotranspiration (mm), average monthly land surface temperature (°C) from eight-day composite, number of monthly malaria cases (2001–2009).

In this study, the dependent variables were monthly time series of malaria case data for the period 2001–2009. Historical cases of malaria and meteorological and environmental variables (rainfall, NDVI, EVI, LST, and ETa) exhibited considerable seasonality and inter annual variation during the period 2001–2009 (Figure 2). A separate time series analysis was conducted for each of the 12 districts of the Amhara region. Because the case data were skewed, log-transformed counts of malaria outpatient cases were used as the dependent variables to linearize relationships with the independent variables. We first developed seasonal autoregressive integrated moving average (SARIMA) models with only malaria cases; in these models, the response depended on past numbers of malaria cases. To test the statistical associations of meteorological and environmental variables with malaria risk, multivariate SARIMA models were developed in which environmental covariates were also included as input variables. In these models NDVI, EVI, rainfall estimate, ETa, and LST were also used as independent variables in these models (Figure 2). However, only rainfall, LST, EVI, and ETa were left in the final models after model selection procedures.

The structure of the model that was used in this study is SARIMA (p,d,q)(P,D,Q)_s_, where p is order of autoregression; d, degree of differencing; q, order of moving average; P, seasonal autoregression; D, degree of seasonal differencing; Q, seasonal moving average; and s, seasonal period. Because stationarity (constant mean and variance) of a time series is a requirement for fitting ARIMA models, first order and seasonal differencing were used to transform the data in to a stationary series. The multivariate ARIMA equation can be written expressing *Y*_*t*_ as a function of its own past values, past errors and lagged independent variables.

$$Y_{t}=\sum_{i=1}^{p}\phi_{i}Y_{t-i}+\sum_{i=1}^{q}\theta_{i}{ɛ}_{t-i}+\sum_{i=1}^{b}\gamma_{i}d_{t-i}$$

where ϕ, θ and γ are coefficients of the autoregression, moving average, and independent variables respectively; *p*, *q*, and *b* are the numbers of past values of $Y_{t-i}$, error terms of ${ɛ}_{t-i}$ and lags of independent variables *d*_*t-i*_ respectively.

First, different SARIMA model forms (different combinations of p, d, q, P, D, and Q) were tested to fit the log-transformed time series data without environmental covariates. The best SARIMA model was selected with the lowest Akaike Information Criterion (AIC), a measure of the relative goodness of fit of a model, across the 12 sites. Second, using the best SARIMA model form, multivariate SARIMA models were fitted with log-transformed malaria cases as response and all the environmental variables with lags ranging from one to three months as potential independent variables. Backward elimination was used to select environmental variables and their associated lags. Environmental variables with p-values less than 0.05 were included in the final model.

These procedures resulted in two final models for each district: a SARIMA model with autoregressive and moving average terms but without environmental variables (Model I), and a multivariate SARIMA model that included autoregressive and moving average terms along with environmental variables (Model II). Data from 2001–2008 were used to estimate model parameters. The fits of these final models (Model I and Model II) were assessed using root mean square error (RMSE) metrics and Akaike weights (w). Akaike weights (w) are based on the AIC statistic and are interpreted as the probability that Model I or Model II is the best approximating model given the data and set of candidate models [27]. The last 12 months of the time series were withheld from model fitting and used to make a one-step-ahead forecast. The observed values of historical case data at lags of one month and one year were used to make predictions for Model I; whereas, observed values of historical case data at lags of one month and one year and environmental variables ranging from lags of one to three months were used to make predictions for Model II. The differences between observed and predicted case numbers were evaluated using the RMSE scores.

All statistical modelling was conducted using SAS software, Version 9.2 of the SAS System for Windows (SAS Institute, Inc., Cary, NC, USA). All geographic data management and processing was carried out using ArcGIS 10 (ESRI, Redlands CA, USA).

## Results

**Figure 3:**
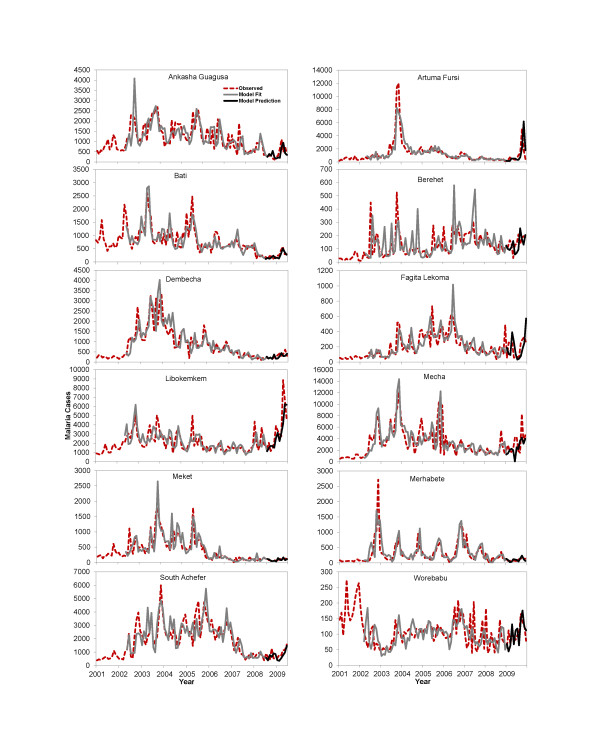
**Observed *vs *fitted and predicted malaria cases from the SARIMA model including environmental covariates (Model II).** Models were fitted using data from 2001–2008 and predictions were made for the last 12 months of the time series.

Outbreaks of malaria cases were observed in 2003, 2005, and 2009 across the 12 sites. While several sites (Bati, Dembecha, South Achefer, Artuma Fursi, Berehet, Mecha, and Merhabete showed peak cases in the year 2003, only Bati, Mecha, and South Achefer exhibited large peaks in 2005 (Figure 3). An increase in malaria cases was also observed in 2009 in several districts. However, no major peaks of cases were observed for Ankasha Guagusa, Fagita Lekoma, Meket, and Worebabu in 2009 (Figure 3).

The best time series regression model, based on AIC values, was found to be a SARIMA (1,1,0)(0,1,1)_12_ since it exhibited the lowest AIC across the 12 study sites. This model form includes the previous month’s malaria cases (p = 1), and a seasonal moving average term (Q = 12), along with first order (d = 1) and seasonal differencing (D = 12). Environmental variables that were included in the final models as predictors were rainfall, LST, EVI, and ETa. Six sites (South Achefer, Mecha, Ankasha Guagusa, Worebabu, Merhabete, and Artuma Fursi) exhibited statistically significant positive relationships of LST with malaria cases at a time lag of one month (Table 1). Rainfall was positively associated with malaria cases at five of the sites (South Achefer, Ankasha Guagusa, Berehet, Bati, and Meket) with a time lag of one to three months. Moreover, the EVI exhibited a positive relationship with a time lag of three months at three study sites (Dembecha, Ankasha Guagusa, and Fagita Lekoma). ETa also showed significant positive relationships with a time lag of one to three months at three of the sites (Libokemkem, Artuma Fursi, and Worebau). However, the NDVI did not exhibit a significant relationship at any study site. Malaria cases exhibited lagged positive relationships with both temperature (LST) and moisture (TRMM rainfall, EVI, and ETa) variables (Table 1). The signs of these coefficients were consistent across the 12 study sites.

**Table 1:** Regression coefficients for Model II across the 12 districts

**Districts**	**Variables**	**β**	**S.E.**	**p**
Ankasha Guagusa	Rainfall at 1-month lag	0.001	0.001	0.027*
	EVI at 3-months lag	4.305	1.467	0.003**
	LST at 1-month lag	0.056	0.025	0.026*
Artuma Fursi	LST at 1-month lag	0.071	0.026	0.006**
	ETa at 1-month lag	0.009	0.003	0.021*
	ETa at 3-months lag	0.010	0.003	0.004**
Bati	Rainfall at 3-months lag	0.001	0.001	0.004**
Berehet	Rainfall at 2-months lag	0.002	0.001	0.048*
Dembecha	EVI at 3-months lag	2.744	1.234	0.026*
Fagita Lekoma	EVI at 3-months lag	4.860	2.063	0.018*
Libokemkem	ETa at 3-months lag	0.007	0.003	0.020*
Mecha	LST at 1-month lag	0.094	0.032	0.004**
Meket	Rainfall at 3-months lag	0.004	0.001	0.002**
Merhabete	LST at 1-month lag	0.128	0.056	0.021*
South Achefer	Rainfall at 3-months lag	0.001	0.001	0.035*
	LST 1-month lag	0.125	0.032	<.0001**
	ETa 1-month lag	0.006	0.002	0.029*
Worebabu	LST at 1-month lag	0.101	0.024	<.0001**
	ETa at 1-month lag	0.0116	0.005	0.042*

* significant at 0.05 level ** significant at 0.01 level.

Adding remotely-sensed environmental variables to the models improved model fit and predictions in 11 of 12 districts, AIC and RMSE scores were lower for Model II than Model I at every site except Libokemkem (Table 2). The Akaike weights for Model II were greater than 0.85 (85%) for all sites except Libokemkem; this result indicated improvement of model fits by adding environmental variables. The fit and prediction accuracy of Model II showed some variability among the 12 study sites (Figure 3). The models fit the historical data well for many districts, including Dembecha, Mecha, and South Achefer. In contrast, model fits under predicted or over predicted peaks in observed cases in other districts, including Berehet, Libokemkem, and Worebabu.

**Table 2:** AIC, RMSE (log-transformed case numbers), and Akaike Weight for Model I and Model II across the 12 districts

**Districts**	**Model I**			**Model II**		
	**AIC**	**RMSE**	**Akaike Weight**	**AIC**	**RMSE**	**Akaike Weight**
Ankasha Guagusa	103.61	0.444	0.002	91.45	0.410	0.998
Artuma Fursi	93.05	0.406	0.080	88.16	0.386	0.920
Bati	74.31	0.378	0.083	69.50	0.360	0.917
Berehet	174.21	0.697	0.033	167.45	0.669	0.967
Dembecha	111.98	0.473	0.051	106.13	0.461	0.949
Fagita Lekoma	132.11	0.514	0.068	126.86	0.505	0.932
Libokemkem	65.66	0.341	0.531	65.91	0.344	0.469
Mecha	125.88	0.492	0.050	120.00	0.471	0.950
Meket	191.19	0.708	0.043	184.97	0.695	0.957
Merhabete	191.99	0.754	0.109	187.78	0.737	0.891
South Achefer	78.44	0.391	0.004	67.16	0.352	0.996
Worebabu	115.13	0.470	0.005	104.39	0.434	0.995

Note: Model I (without environmental covariates); Model II (with environmental covariates).

For the last 12 months of the time series, Model II predicted 104,541 cases across all the study districts; whereas, 122,036 cases were reported. Prediction skill varied among districts ranging from 17% overprediction (modelled cases > actual cases) to 26% underprediction (modelled < actual), but predictions for seven of 12 districts are within 10% of the reported cases (Table 3). In districts in which Model II over or under predicted by more than 10% (Bati, Dembecha, Libokemkem, South Achefer, and Mecha), other factors not included in the model likely also influenced malaria risk.

**Table 3:** Summary of prediction accuracy for the last 12 months of the time series for Model II across the 12 districts

**District**	**Predicted Cases**	**Reported Cases**	**Delta**	**% Difference**
Bati	3149	2697	452	16.8
Ankasha Guagusa	13711	12805	906	7.1
Fagita Lekoma	2310	2199	111	5.0
Merhabete	1226	1193	33	2.8
Worebabu	1228	1217	11	0.9
Meket	1050	1057	−7	−0.7
Artuma Fursi	4695	5142	−447	−8.7
Berehet	1576	1739	−163	−9.4
Dembecha	3266	3649	−383	−10.5
Libokemkem	35062	41371	−6309	−15.2
South Achefer	8654	10260	−1606	−15.7
Mecha	28614	38707	−10093	−26.1

## Discussion

Rainfall was associated with malaria cases at a lag of one to three months in five of the study sites. This result is consistent with previous studies. A study in neighbouring Eritrea showed a correlation of satellite-based rainfall estimates with two to three months lead time and malaria cases [24]. The current findings are also consistent with a study in Ethiopia, which demonstrated that rainfall measurements from meteorological stations were significantly associated with malaria cases in lags of 10–12 weeks in highland districts and six to 10 weeks in lowland districts, respectively [5]. Another study in Ethiopia showed that there was a strong association of rainfall based on meteorological station data with malaria at a lag of two to three months [13]. A study in Rwanda similarly demonstrated that malaria incidence was significantly associated with rainfall [28]. The current research extends these previous studies by demonstrating that remotely sensed precipitation estimates can be used to model temporal patterns of malaria cases. It should be noted that the accuracy of satellite precipitation estimates are affected by sampling and spatial and temporal heterogeneity such as topography, location, and rainfall type. However, a previous study found that TRMM based precipitation estimates over the complex terrain of the Ethiopian highlands outperformed other satellite rainfall products [18].

The relationship between air temperature measured at meteorological stations and malaria risk has been highlighted in several previous studies [14,29], including studies of climatic variability and malaria risk in Ethiopia [5,16]. For example, one of these studies found positive significant associations of minimum temperature with malaria cases in the highland regions of Ethiopia at lags ranging from seven to 10 weeks [5]. In this study, LST was positively associated with malaria incidence at a lag of one month in six districts. LST is estimated from thermal radiance that is emitted from the land surface [30]. Satellite-based measurements of LST are sensitive to thermal characteristics of the ground, atmospheric effects of spectral radiation and bulk emissivity of the mixture of materials within the scene. As a result, temporal patterns of LST may not be tightly correlated with the near surface air temperature [31]. Despite these caveats, results indicate that LST is a potential indicator of malaria risk in the highlands of Ethiopia. In the current application, LST was suitable for prediction because despite its limitations, it captured relative values of temperature through time and among sites.

Vegetation greenness is expected to respond positively to rainfall in moisture-limited regions of East Africa; thus, it could serve as an indirect indicator of surface water and near-surface humidity. Several other studies have reported positive associations of vegetation indices and malaria risk [24,32,33]. For example, a study in Eritrea showed association of NDVI with malaria at a lag of four months [24]. In the present study, there was significant association of EVI and malaria risk in three districts at a lag of three months. In contrast, NDVI exhibited no significant association with malaria cases in the final models. Although the NDVI is a widely used vegetation index that can provide insights into vegetation dynamics, it loses sensitivity over denser vegetation [34-37]. The EVI offers an alternative index that retains more sensitivity than the NDVI over denser vegetation, thereby enabling it to capture more variation and change in a mature canopy [34,35,37]. Land cover across the Amhara region is mostly cropland and herbaceous vegetation, and a comparison of NDVI and EVI time series exhibits the expected loss of sensitivity of NDVI at higher EVI values (data not shown).

Findings from this study show novel results on the lagged positive association between ETa and malaria cases. No other study has examined the temporal relationship between ETa and malaria occurrence. Results showed a significant positive association of ETa and malaria cases in three of the 12 study sites at lags of one to three months. ETa provides information on the flux of moisture at and below soil, plants, and water bodies, and is therefore an indicator of water availability at the soil surface. Thus, ETa may offer a more proximal measurement of environmental conditions suitable for mosquito breeding and can provide a new avenue for monitoring environmental risk factors for malaria and, perhaps, other vector borne diseases.

Although this study has practical utility towards developing a malaria early-warning system, several limitations should be noted. First, the current models include only remotely sensed environmental indicators. However, there are other confounding factors that affect malaria risk such as land use/land cover, population mobility, local hydrology, socio-economic factors, and public health interventions; these processes are not captured in the current model. Additional research will be necessary to quantify these influences and incorporate them into future modelling efforts. Second, it should be noted that this type of time-series model is strongly data-driven, requiring a sufficient time series of historical data for model parameterization. Furthermore, consistent collection and timely reporting of malaria surveillance data, preferably at weekly rather than monthly intervals, would be needed to enable operational forecasting using this type of autoregressive model, which includes surveillance case data as predictor variables.

More specifically, the univariate SARIMA modelling approach (Model I) can be viewed as a type of malaria early-detection strategy, which uses temporal patterns of historical cases as a baseline to identify anomalies that may indicate the early stages of an emerging epidemic [38,39]. As a type of early detection, Model I can use historical case data to make short-term forecasts of whether malaria cases are likely to increase or decrease in upcoming months. In contrast, disease-forecasting efforts based on environmental data alone are typically referred to as early warning [5-7]. The potential for combining early detection and early warning to leverage the advantages of both approaches has not been widely recognized or explored. The improved fit of Model II highlights the feasibility of this blended approach by demonstrating that predictions from early detection models can be improved by incorporating remotely sensed environmental variables.

Overall, temporal patterns of malaria were associated with satellite-derived meteorological and environmental variables. However, there is spatial variation in the impact of environmental drivers across the study sites. This spatial variation may reflect other factors such as differences in land use/land cover, local hydrology, topography, immunity of the population, population movement, vector control, and health access. Moreover, there was a geographical pattern of the statistically significant predictor variables in the models. For instance, malaria was associated with vegetation (EVI) in three neighbouring sites (Ankasha Guagusa, Dembecha, and Fagita Lekoma) on the southwest of the Amhara region. On the other hand, LST was found to be a statistically significant predictor of malaria risk across broader geographic areas. Thus, there is a potential for identifying geographical areas where similar environmental drivers predict malaria risk. The resulting information could be used to spatially stratify the region and develop models for malaria early warning that are tailored to specific areas.

Prediction skill varied among the districts. The results and their potential application for operational early warning system should therefore be interpreted cautiously. It should also be noted that there are limitations to the clinically diagnosed malaria data due to inconsistent registration of cases, incomplete reporting, and lack of centralized data sharing platforms. In some cases, patients visiting a health centre may come from neighbouring districts and may not be exposed to the same climate and environment. These uncertainties likely contributed to the variable levels of error in the current model predictions. To set up a robust malaria early warning system, there is a need for routine surveillance data at weekly temporal resolution and district level that is disseminated in a digital format. The current study contributes to quantifying the association of climatic and environmental variability with malaria risk by using remotely sensed variables and historical morbidity data in an effort towards the development of a malaria early-warning system in the Amhara region.

## Conclusions

Development of operational Malaria Early Warning Systems (MEWS) has been proposed by the World Health Organization (WHO) to combat malaria epidemics particularly in climate sensitive regions [7]. In particular, an integrated malaria early-warning system can be developed using environmental monitoring and epidemic surveillance. One of the key steps to develop effective malaria early warning is to quantify the relationship between malaria cases and meteorological and environmental determinants. In this study a time series regression SARIMA modelling approach was used to quantify the lagged association of environmental variables with malaria cases and predict malaria cases among 12 districts in the Amhara Region of Ethiopia. The major finding in the current study was that there was a strong lagged association between malaria cases and satellite-derived meteorological and environmental variables across the 12 sites in the Amhara region of Ethiopia. This lead time of one to three months can be used to develop a malaria early-warning system for the region. Equally important, this study highlighted the potential for integrating modelling approaches based on historical case data (early detection) and environmental data (early warning) to enhance the effectiveness of malaria risk forecasting efforts.

## Competing interests

The authors declare that they have no competing interests.

## Authors' contributions

AM and MCW designed the study, processed the satellite data, analysed the data, and drafted the manuscript; GMH participated in satellite data processing and drafting the manuscript. GS also participated in drafting the manuscript. PS developed the historical malaria surveillance database. The authors all read and approved the manuscript.
